# PAQR-2 Regulates Fatty Acid Desaturation during Cold Adaptation in *C. elegans*

**DOI:** 10.1371/journal.pgen.1003801

**Published:** 2013-09-12

**Authors:** Emma Svensk, Marcus Ståhlman, Carl-Henrik Andersson, Maja Johansson, Jan Borén, Marc Pilon

**Affiliations:** 1 Department of Chemistry and Molecular Biology, University of Gothenburg, Gothenburg, Sweden; 2 Department of Molecular and Clinical Medicine/Wallenberg Laboratory, Institute of Medicine, University of Gothenburg, Gothenburg, Sweden; University of California San Francisco, United States of America

## Abstract

*C. elegans* PAQR-2 is homologous to the insulin-sensitizing adiponectin receptors in mammals, and essential for adaptation to growth at 15°C, a low but usually acceptable temperature for this organism. By screening for novel *paqr-2* suppressors, we identified mutations in genes involved in phosphatidylcholine synthesis (*cept-1, pcyt-1 and sams-1*) and fatty acid metabolism (*ech-7*, *hacd-1*, *mdt-15*, *nhr-49* and *sbp-1*). We then show genetic evidence that *paqr-2*, phosphatidylcholines, *sbp-1* and Δ9-desaturases form a cold adaptation pathway that regulates the increase in unsaturated fatty acids necessary to retain membrane fluidity at low temperatures. This model is supported by the observations that the *paqr-2* suppressors normalize the levels of saturated fatty acids, and that low concentrations of detergents that increase membrane fluidity can rescue the *paqr-2* mutant.

## Author Summary

Cold-blooded organisms such as insects, fish or worms must make physiological adjustments when the temperature in their environment decreases. One essential adaptive measure is to increase the fluidity of the cellular membranes that are made of fatty molecules and would tend to harden at low temperatures, just as butter would. In our study we identify genes that are regulated by PAQR-2, a membrane protein that we show to be essential for adjusting the membrane fluidity during cold adaptation in the nematode *C. elegans*. Interestingly, the genes influenced by PAQR-2 are all involved in fatty acid metabolism. We speculate that the human homologs of PAQR-2, which are receptors for the hormone adiponectin, may have similar functions.

## Introduction

To function over a range of temperatures, poikilotherms must make adaptive adjustments to their physiology [1]–[4]. For example, adaptation to cold in *Drosophila* correlates with changes in the phospholipid composition of cellular membranes, favoring a higher abundance of shorter and/or unsaturated fatty acids to maintain membrane fluidity [5]–[8]. Another aspect of cold adaptation involves the activation of stress responses, including the inaptly named heat shock proteins [4], [9], [10]. On the whole however, little is known about the regulatory pathways, from receptors to effectors, that coordinate the well-documented physiological changes that poikilotherms make as they adapt to cold temperatures.

The *C. elegans paqr-2* mutant is unable to adapt to growth at 15°C, which is close to the lowest temperature at which this species can be propagated [11], [12]. *paqr-2* encodes a protein with seven transmembrane domains homologous to the mammalian adiponectin receptors, AdipoR1 and AdipoR2, that are insuling-sensitizing metabolic regulators of which the signaling pathways are not well understood [1]–[3], [13]–[15]. Like its mammalian homologs, *paqr-2* is important for metabolic regulation: the *paqr-2* mutant shows an abnormal fatty acid (FA) composition, an excess of lipid droplets when combined with a *paqr-1* mutation, and is synthetic lethal with loss-of-function (*lof*) mutations in genes that promote FA turnover such as *sbp-1* and *nhr-49* [5], [12], which are *C. elegans* homologs of SREBP and HNF4 [16]–[19]. One important limitation of our earlier work on *paqr-2* is that we used a candidate gene approach, mostly inspired from previous studies in mice, to try and identify genes that may interact with *paqr-2*. While often successful, such an approach makes it impossible to discover unexpected interactions. Here, we used an unbiased forward genetic approach in which we screened randomly mutagenized worm populations to isolate and then characterize mutations that suppress the cold adaptation defect of the *paqr-2* mutant. This is a classical investigative strategy for which *C. elegans* is eminently suited [20]. Using this approach, we isolated nine *paqr-2* suppressors and establish a novel regulatory pathway connecting *paqr-2*, phosphatidylcholine (PC) synthesis and the regulation of FA desaturation to increase membrane fluidity during cold adaptation.

## Results

### The *paqr-2(tm3410)* mutant

The *paqr-2(tm3410)* allele carries a deletion that eliminates the first transmembrane domain and introduces a premature stop codon prior to all remaining transmembrane domains [12] (Figure S1A). Consistently, antibodies raised against the C-terminus of PAQR-2 detect a band of ∼66 kDa that is absent in the *paqr-2(tm3410)* mutant but reappears when the wild-type *paqr-2* gene is reintroduced as a transgene (Figure S1B). *paqr-2(tm3410)*, later referred to simply as *paqr-2*, is therefore likely a null allele.

### A *paqr-2* suppressor screen

**Figure 1:**
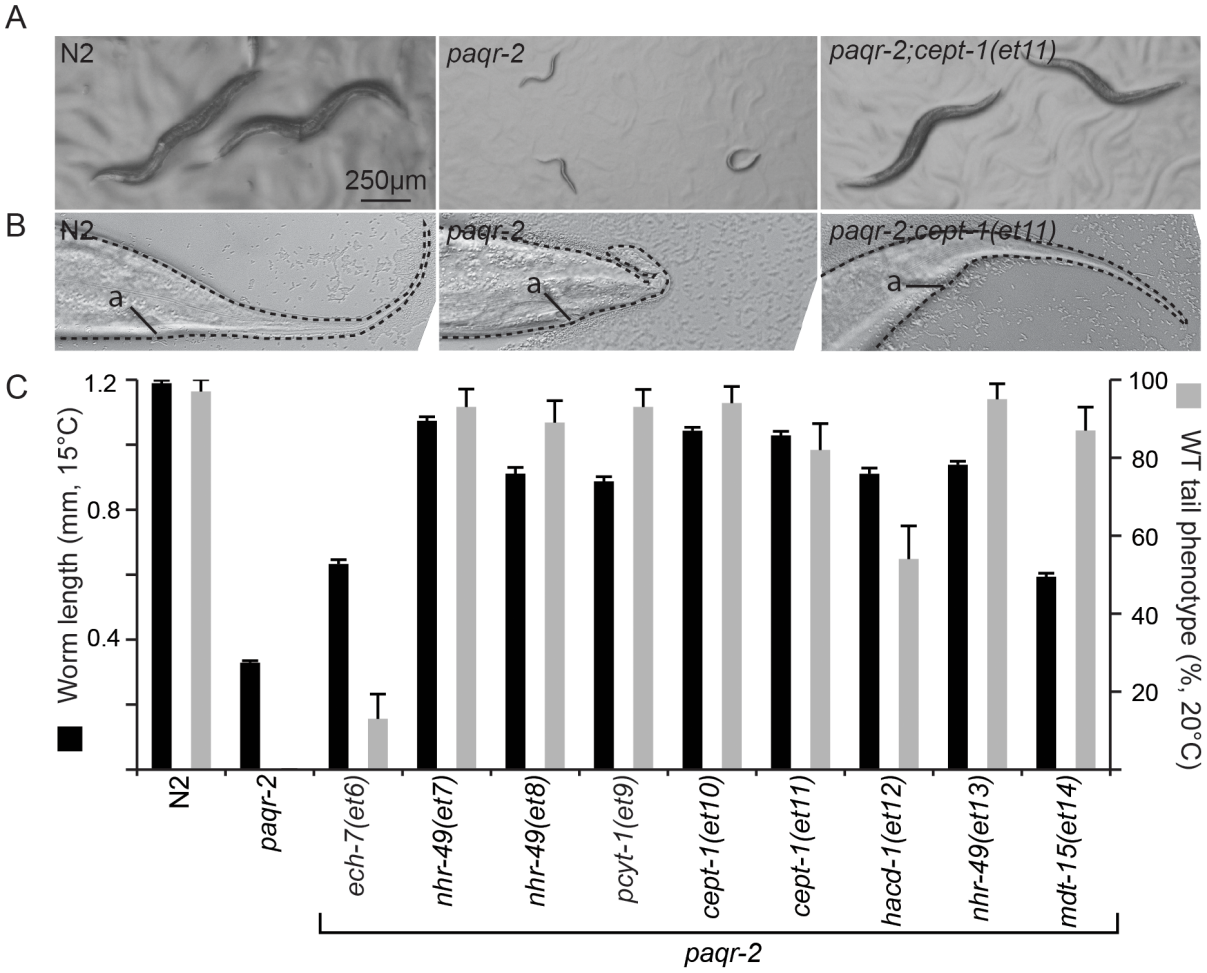
Overview of the *paqr-2* suppressor mutations. (**A**) Wild-type (N2), *paqr-2* mutants and *paqr-2* mutant carrying the suppressor mutant allele *et11* photographed after 144 hours of cultivation at 15°C. (**B**) Tail of wild-type (N2), *paqr-2* mutant and *paqr-2* mutant carrying the suppressor mutant allele *et11* grown at 20°C. “a” indicates the position of the anus. (**C**) Quantification of the suppression of two *paqr-2* phenotypes by the nine isolated suppressor alleles (*et6–et14*).

We mutagenized *paqr-2* mutant worms and screened the F2 progeny for their ability to grow and reproduce at 15°C. A screen of ∼15 000 mutagenized haploid genomes led to the isolation of 9 *paqr-2* suppressor mutants (alleles *et6-et14*; Figure 1A and C; Figure S2A) all of which also suppressed the characteristic *paqr-2* withered tail tip phenotype (Figure 1B and C), as well as brood size and length defects at 20°C (one exception is the *et6* allele which only slightly rescued the tail phenotype, did not rescue brood size and caused a slight reduction in length at 20°C; Figure S2B–C). The suppressor mutations therefore suppress multiple aspects of the *paqr-2* mutant phenotype, rather than merely improving cold-adaptation.

### *paqr-2* suppressors

**Figure 2:**
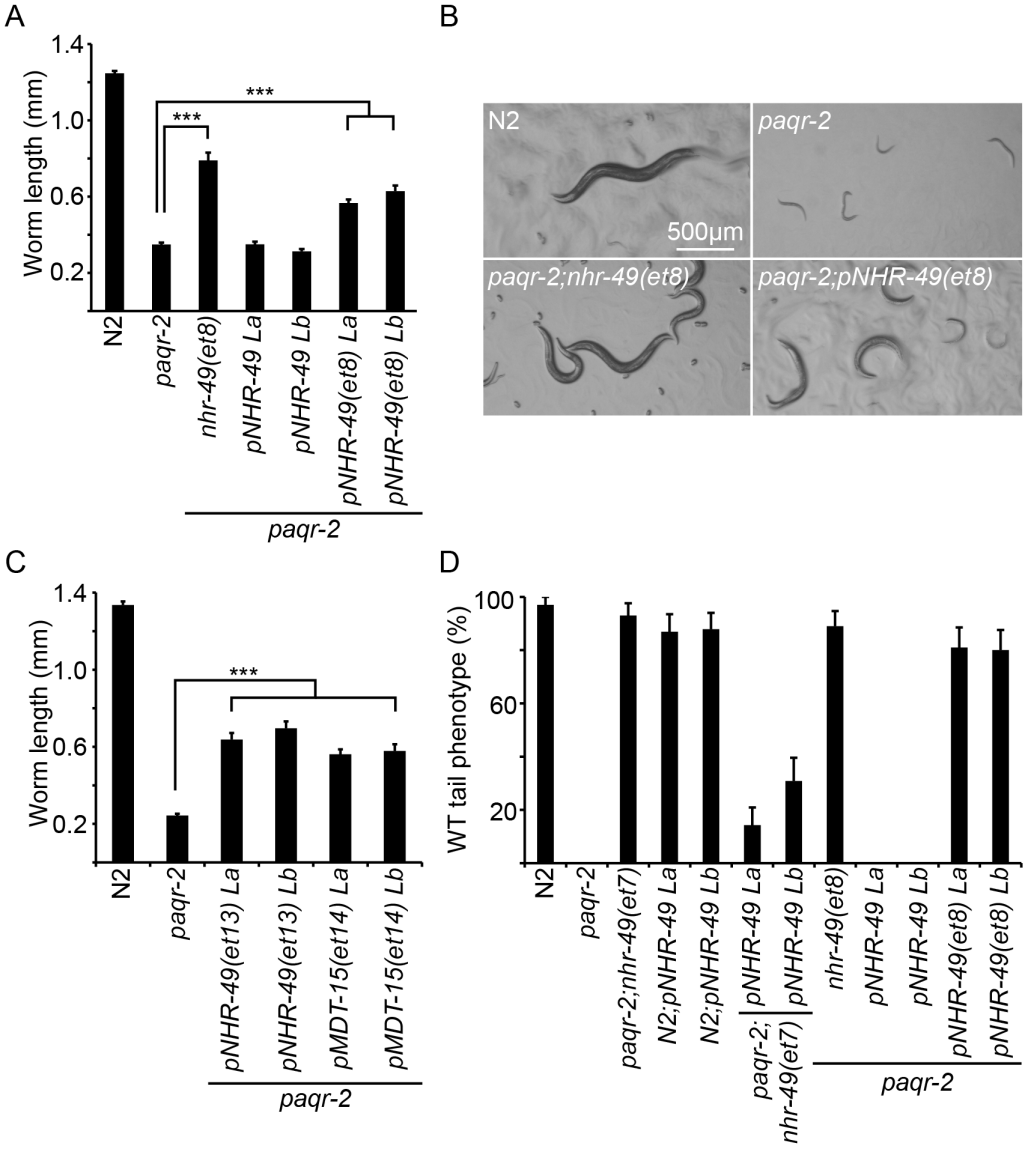
The *paqr-2* suppressor mutations in *nhr-49* and *mdt-15* are *gof* alleles. (**A**) Length of worms grown for 144 hours at 15°C. Results from two *paqr-2* lines carrying the plasmids *pNHR-49* and *pNHR-49(et8)* are shown; only strains carrying *pNHR-49(et8)* showed improved growth indicating that it is a *gof* allele. (**B**) Representative images from four of the genotypes used in panel A. (**C**) Providing *nhr-49(et13)* or *mdt-15(et14)* as transgenes suppress the 15°C growth defect of the *paqr-2* mutant, indicating that they are *gof* alleles. The results from two lines are shown for each transgene. (**D**) Homozygosity for the *nhr-49(et7)* or *nhr-49(et8)* mutations suppress the *paqr-2* withered tail tip phenotype. Providing *nhr-49(et8)* as a transgene also suppresses the *paqr-2* the tail tip defect, again demonstrating that *et8* is a *gof* allele. ***: *p*<0.001.

**Figure 3:**
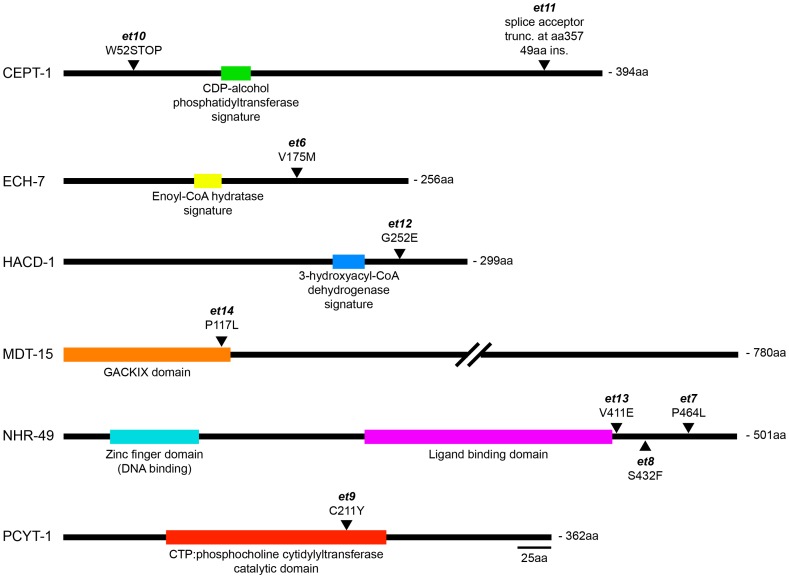
The novel *paqr-2* suppressor mutations affect proteins that regulate lipid metabolism. For each protein, the position and nature of the novel *paqr-2* suppressor alleles are indicated as well as the position of important functional domains.

**Figure 4:**
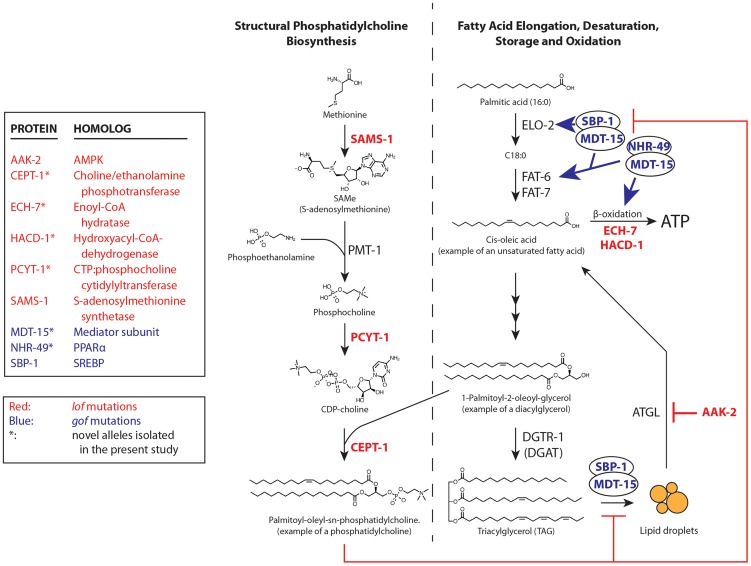
*paqr-2* suppressors either inhibit phosphatidylcholine synthesis or promote fatty acid metabolism. Red- and blue-labeled names indicate proteins where *lof* and *gof* alleles can suppress the *paqr-2* phenotypes, respectively; for *sbp-1* we used a multicopy transgene as a *gof* allele. The *nhr-80(tm1011) lof* allele also suppresses the *paqr-2* cold adaptation defect [12]; it is omitted from the figure for clarity.

To molecularly define the suppressor mutations, we used a strategy based on back-crossing and whole-genome sequencing [21], [22]: each suppressor was outcrossed 4 or 6 times to wild type and *paqr-2* single mutants, and its genome was then sequenced. For each suppressor, mutation clusters present in the outcrossed strains defined a region of interest in which to look for the suppressor mutation. Candidate mutations were tested using three strategies: 1) Attempt to desuppress the *paqr-2* phenotypes by introducing the wild-type version of the candidate gene into the suppressor strain; 2) Test whether available loss-of-function (*lof*) mutations or RNAi inhibition of the candidate gene suppress the *paqr-2* phenotypes; and 3) When a gain-of-function (*gof*) mutation was considered, attempt to suppress the *paqr-2* phenotypes by introducing the mutated version of the candidate gene as a transgene. Figures 2 and S3 show the experimental data confirming the identification of the *paqr-2* suppressors, Figure 3 and Table 1 describe their molecular nature, and Figure 4 presents them in the form of a metabolic/genetic pathway.

**Table 1:** Genes that can be mutated to suppress the *paqr-2* cold adaptation defect.

Gene	Allele/transgene	Type	Molecular description	Flanking sequence (25 bp, (+) strand)	Experimental evidence for *paqr-2* supp ressive activity
*aak-2*	*ok524*	Lof	X: 408 bp deletion	L: agtacaccttctgacattttcatgR: tagaaatccaattaaagtaattgaa	Introduction of *aak-2(ok524)* partially suppresses the 15°C growth defect of *paqr-2* [12]
*cept-1*	*et10*	lof	X: G12763193A; W52STOP	L: tttatcgatgtacgtttttcagatgR: tgggagtttgtgattacattatgcc	Introduction of WT *cept-1* as a transgene in *paqr-2;cept-1(et10)* desuppresses the *paqr-2* tail defect at 20°C and brood size at 15°C (Figure S3C–D)
*cept-1*	*et11*	lof	X: G12765379A, splice acceptor	L: gtatattttacttcatccactttcaR: atatactgttatacttctcttttca	Introduction of WT *cept-1* as a transgene in *paqr-2;cept-1(et11)* desuppresses the *paqr-2* tail defect (Figure S3D)
*ech-7*	*et6*	lof	I: C14368566T; V175M	L: tcaggaggcgaaggaagacggcctgR: tgagcaaggtgttcccggtgcagca	Injected *ech-7* RNAi suppresses the 15°C growth defect of *paqr-2* (Figure S3A)
*hacd-1*	*et12*	lof	V: G1461311A; G252E	L: ccaattgaattgtgtgactatgttgR: acttgatgttctgcagagcactttg	Introduction of *hacd-1(ok2776)* suppresses the 15°C growth defect of *paqr-2*. Introduction of WT *hacd-1* as a trasgene in *paqr-2;hacd-1(et12)* desuppresses the *paqr-2* tail defect (Figure S3B and D)
*mdt-15*	*et14*	gof	III: C5832666T; P117L	L: gctcctgtgcctccagatccacaacR: aacatcagctcaggcaagaaatcca	Introduction of *mdt-15(et14)* as a transgene suppresses the 15°C growth defect of *paqr-2* (Figure 2C)
*nhr-49*	*et7*	gof	I: C9874044T; P464L	L: atgctctcagcaactcttgcagctcR: attggcaattcatcctctccaatca	Introduction of WT *nhr-49* as a transgene in *paqr-2;nhr-49(et7)* desuppresses the *paqr-2* tail defect (Figure 2D)
*nhr-49*	*et8*	gof	I: C9873765T; S432F	L: aatgatcattcgacggctccggtctR: tttacagcaacatctttcatctccg	Introduction of *nhr-49(et8)* as a transgene suppresses the tail and 15°C growth defect of *paqr-2* (Figure 2A and D)
*nhr-49*	*et13*	gof	I: T9873702A; V411E	L: tccgtacataaatgatagcttcaggR: ggattctctgctctccgagttcatt	Introduction of *nhr-49(et13)* as a transgene suppresses the 15°C growth defect of *paqr-2* (Figure 2C)
*nhr-80*	*tm1011*	lof	III: 446 bp deletion	L: tttattagaaaactcacacaatgctR: gtctgcggggacacattcgcgaatt	Introduction of *nhr-80(tm1011)* partially suppresses the 15°C growth defect of *paqr-2* [12]
*pcyt-1*	*et9*	lof	X: G7579636A; C211Y	L: ggtgtctctacaagtgacgtcgtctR: cagaatcatccgtgattacgataag	Injected *pcyt-1* RNAi partially suppresses the 15°C growth defect of *paqr-2* (Figure S3A)
*sams-1*	*ok2946*	lof	X: 707 bp deletion	L: gtttcaatgtttttgttcaggattR:tcggaacttccccactcaccgacga	Introduction of *sams-1(ok2946)* suppresses the 15°C growth defect of *paqr-2* (Figure S3E)
*sbp-1*	*epEx141*	Over exp.	sbp-1::GFP::SBP-1+rol-6(su1006)	Extra chromosomal array	Introduction of *epEx141* suppresses the 15°C growth defect of *paqr-2* (Figure S3F)

The *et6* to *et14* alleles were isolated in this study. Genomic positions refer to Wormbase release WS200. The *aak-2* and *nhr-80* alleles were identified as *paqr-2* suppressors in an earlier study [12].

Briefly, three *paqr-2* suppressors are *gof* alleles of *nhr-49* (alleles *et7*, *et8* and *et13*; Figure 2), and a fourth is a *gof* mutation in *mdt-15* (allele *et14*; Figure 2C). *nhr-49* encodes a nuclear hormone receptor homologous to the mammalian HNF4 and with activities that are sometimes compared to that of PPARα, while *mdt-15* encodes a subunit of Mediator, an RNA polymerase II transcription co-regulator, that interacts with the NHR-49 and SBP-1 transcription factors to promote FA desaturation, elongation and β-oxidation [17], [23]–[25]. Several lines of evidence support the conclusion that these *paqr-2* suppressors are *gof* alleles: 1) They act in a dominant fashion when introduced as multicopy transgenes into *paqr-2* mutant worms (Figure 2); 2) The *nhr-49(et8)* acts as a dominant allele in a standard genetics assay whereby worms heterozygous for this allele but homozygous for the *paqr-2* mutation were scored for growth at 15°C (data: 59.5% of F2 progeny from *paqr-2;nhr-49(et8)*/+ mothers grew into adults at 15°C, which matches the 62.5% expectation if *nhr-49(et8)* is dominant rather than the 37.5% if it was recessive; n = 341); and 3) The *nhr-49(et8) gof* allele suppresses *paqr-2* phenotypes while the *nhr-49(gk405) lof* allele is synthetic lethal with *paqr-2*, and these two alleles have opposite effects on fatty acid composition and *fat-7* regulation (see below).

Two other *paqr-2* suppressor mutations are *lof* alleles of the genes *ech-7* (allele *et6*; Figure S3A) and *hacd-1* (allele *et12*; Figure S3B and D). These genes encode the worm homologs of enoyl-CoA hydratase and of hydroxyacyl-CoA dehydrogenase, which perform consecutive reactions during FA β-oxidation in mitochondria.

The remaining three *paqr-2* suppressor mutations are *lof* alleles of *pcyt-1* (allele *et9*; Figure S3A) and *cept-1* (alleles *et10* and *et11*; Figure S3C–D), the worm homologs of CTP:phosphocholine cytidylyltransferase and of choline/ethanolamine phosphotransferase, which are enzymes important for PC synthesis [19]. While our experimental evidence show that *pcyt-1(et9)* is a *lof* allele, it may be a hypomorph rather than a null allele since homozygosity for the *pcyt-1(ok547)* deletion allele is reported in Wormbase to cause lethality. Note however that the *pcyt-1(ok547)* allele is unpublished and may not have been thoroughly outcrossed.

The newly isolated *paqr-2* suppressors, as well as the previously identified partial suppressors *aak-2(ok524)* and *nhr-80(tm1011)* [12], are all involved in PC synthesis and FA metabolism. Building on these findings, we also tested other genes involved in these processes and found that a *sams-1 lof* mutation can suppress the cold adaptation defect of *paqr-2* mutants (Figure S3E). *sams-1* encodes S-adenosylmethionine synthetase that converts methionine to S-adenosylmethionine, the methyl donor during phosphocholine synthesis [19]. Separately, we found that an overexpression transgene of *sbp-1* can rescue the *paqr-2* cold adaptation defect (Figure S3F). *sbp-1* is an activator of the Δ9 desaturases *fat-5*, *fat-6* and *fat-7* that also regulates lipid storage [23], [26]. In summary, the *paqr-2* suppressor mutations affect genes of two basic metabolic pathways (Figure 4): 1) PC biosynthesis: *sams-1*, *pcyt-1* and *cept-1*; and 2) FA metabolism: *aak-2*, *ech-7*, *hacd-1*, *mdt-15*, *nhr-49*, *nhr-80* and *sbp-1*. Importantly, these two pathways are functionally related: low levels of PCs are associated with activation of *sbp-1*/SREBP in metazoans [19].

### *paqr-2* mutants accumulate saturated fatty acids

**Figure 5:**
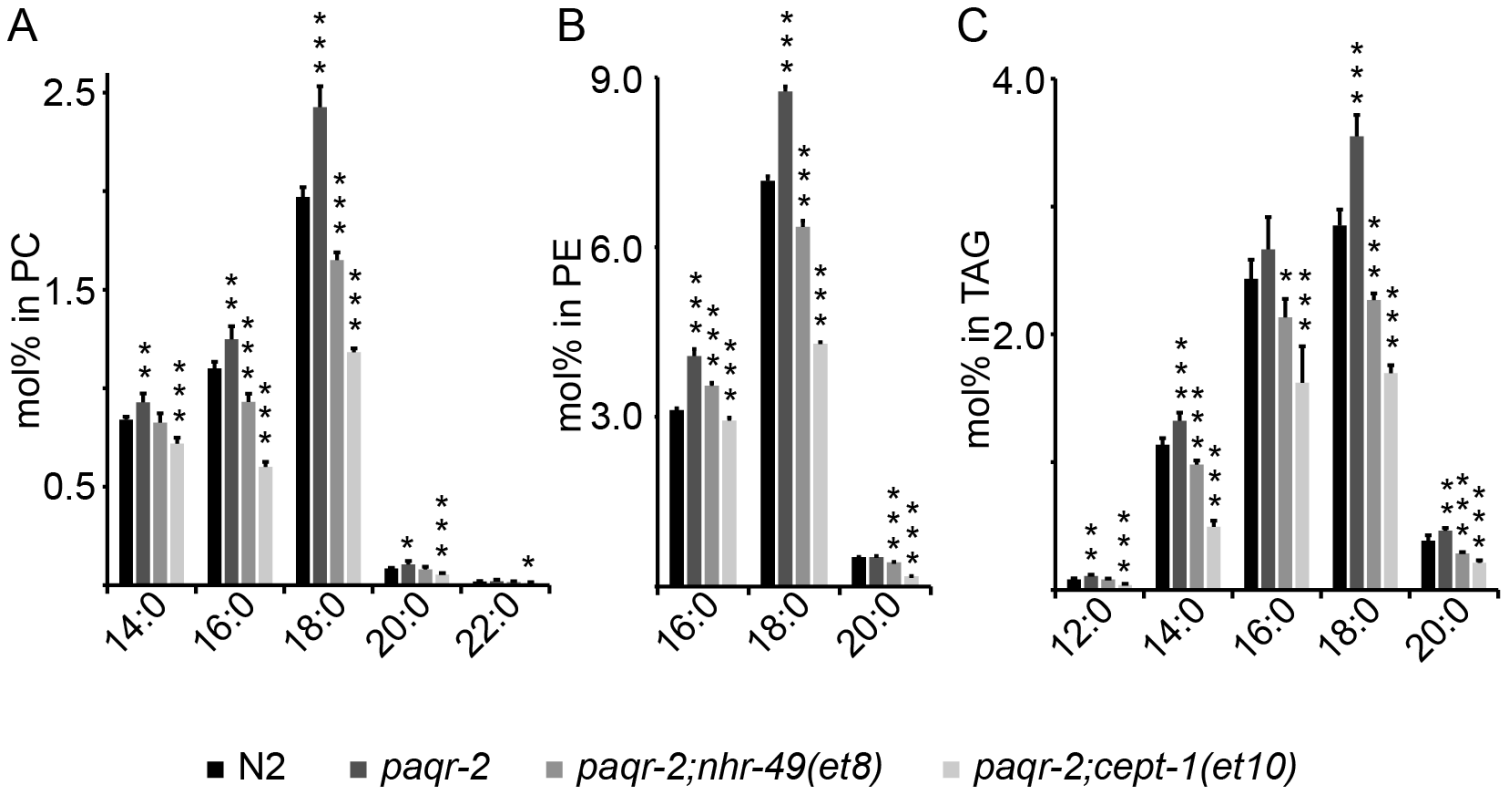
Fatty acid composition defects in the *paqr-2* mutant. The *paqr-*2 mutant has an excess of saturated even-chained FAs in PCs (**A**), PEs (**B**) and TAGs (**C**), and these defects are corrected or overcompensated by the *nhr-49(et8)* and *cept-1(et10)* suppressor mutations. *: *p*<0.05; **: *p*<0.01; ***: *p*<0.001.

Mechanistically, the identity of the *paqr-2* suppressors strongly implicates this transmembrane receptor as a regulator of FA metabolism during cold adaptation and tail tip maintenance. We hypothesized that a common outcome of all the *paqr-2* suppressor mutations is an increase in unsaturated fatty acids. To test this, we determined the FA composition of PCs, phosphatidylethanolamines (PEs) and triacylglycerols (TAGs) in synchronized wild-type, *paqr-2* mutants, and *paqr-2* mutants carrying suppressor mutations. An analysis of 5 biological replicates per genotype showed that 35 of 98 PC species and 19 of 82 PE species are present at significantly elevated levels in the *paqr-2* mutant, and that most of the elevated species carry either one (29/54) or two (10/54) saturated FAs (Tables S1, S2). Similarly, 9 of 13 TAGs that contain two or three saturated FAs were significantly increased in the *paqr-2* mutant (Table S3). Measuring directly the relative abundance of individual FA chains in each of the three lipid types studied showed that saturated even-length FAs are almost all significantly increased in the *paqr-2* mutant and decreased in the *paqr-2;nhr-49(et8)* and *paqr-2;cept-1(et10)* double mutants (Figure 5; Table S4). It is evident that the *paqr-2* mutant has an abnormally high abundance of saturated FAs in all lipid types assayed, and that suppression of the *paqr-2* phenotypes is associated with an increased abundance of unsaturated FAs.

To better describe the consequences of the *nhr-49(et8)* and *cept-1(et10)* mutations, we also analyzed the lipid profiles of these single mutants in comparison with those of wild-type and *paqr-2* mutant worms (Tables S5, S6, S7, S8). The *nhr-49(gk405)* null mutant was also included in this analysis. The results show that the *nhr-49(et8)* and *nhr-49(gk405)* alleles often have opposite effects on the degree of fatty acid desaturation. For example, in PCs the *et8* and *gk405* alleles went 18 times in opposite directions compared to N2 control worms, and only 4 times in the same direction, the trend being a decrease in the proportion of saturated FAs in the *et8* mutant. The effect was also found in the composition of PEs where *et8* and *gk405* went 25 times in opposite directions and 14 times in the same direction. In general, *nhr-49(et8)* had much less even-chained saturated FAs (16∶0, 18∶0 and 20∶0) than *nhr-49(gk405)*, and much more 18∶2 and 20∶2 in all three lipid classes. These results are consistent with the two alleles having opposite effects on the activity of *nhr-49*, with *et8* causing a decrease of saturated fatty acids and *gk405* causing an increase in saturated FAs. As shown in Tables S5, S6, S7, S8, the *cept-1(et10)* mutant is also very enriched in unsaturated FAs, especially in PEs and TAGs, and showed the most depletion of TAGs with no or just a single unsaturated bond. In conclusion, the *nhr-49(et8)* and *nhr-49(gk405)* behave opposite in many lipid measurements, as expected from a gain-of-function vs loss-of-function comparison, and both *nhr-49(et8)* and *cept-1(et10)* tend to have lower levels of saturated FAs, consistent with the hypothesis that they boost the activity of Δ9-desaturases.

Intriguingly, *paqr-2* suppressors normalized the overall PE/PC ratio, which is slightly elevated in the *paqr-2* mutant at 20°C (Figure S4A). This was true even for the *cept-1 lof* alleles, which would be expected to lower PC levels, hence raise the PE/PC ratio. This result suggests the possibility that the *paqr-2* mutant may compensate for an inability to upregulate FA unsaturation by increasing the PE/PC ratio, perhaps by regulating the insertion of dietary lipid types into membranes or by regulating their turnover. Modulating the PE/PC ratio has important effects on membrane dynamics since PEs are bilayer-destabilizing phospholipids [1]. Of the single mutants *nhr-49(et8)*, *cept-1(et10)* and *nhr-49(gk405)*, only the *cept-1* mutant had a PE/PC ratio that differed significantly from wild-type, being elevated as expected from a *lof* allele of *cept-1* given the role of this gene in PC biosynthesis (Figure S4B).

### The *paqr-2* suppressors do not act via *paqr-1*

**Figure 6:**
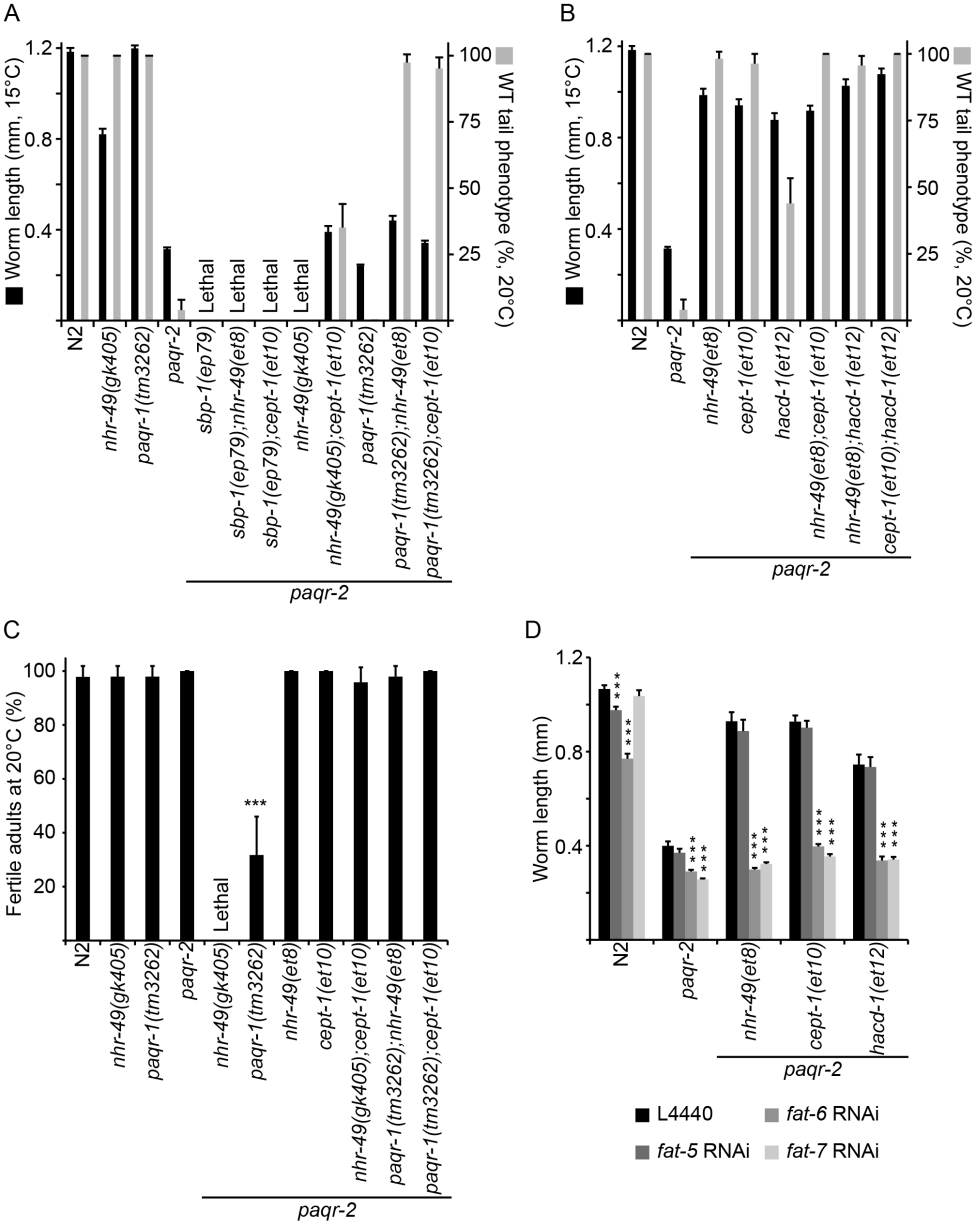
*paqr-1* is not required for *paqr-2* suppression while *sbp-1* and Δ9-desaturases are especially important. (**A**) and (**B**) Length of worms after cultivation of L1s at 15°C for 144 hours. Note that *paqr-1* is not essential for the ability of *nhr-49(et8)* or *cept-1(et10)* to suppress the tail phenotype at 20°C. Also, the *paqr-2;nhr-49(gk405)* synthetic lethality is suppressed by *cept-1(et10)*, while the *paqr-2 sbp-1(ep79)* synthetic lethality is not. (**C**) Fertility at 20°C. Note that the *nhr-49(et8)* and *cept-1(et10)* alleles are able to restore high fertility to the *paqr-1;paqr-2* double mutants, indicating again that their effects are independent of *paqr-1*. Also, note that *cept-1(et10)* completely suppresses the synthetic lethality of *paqr-2;nhr-49(gk405)*, demonstrating that *et10* does not act via *nhr-49*. (**D**) The *paqr-2* suppressor effects of *nhr-49(et8)*, *cept-1(et10)* and *hacd-1(et12)* are abolished when *fat-6* or *fat-7* are inhibited by RNAi while the control L4440 RNAi vector or RNAi against *fat-5* has no effect.

*paqr-1* shows sequence homology to *paqr-2*, and although the *paqr-1* mutant has neither cold adaptation or tail tip morphology defects, there is some functional overlap between the two genes [12]. It was therefore important to explore the possibility that the *paqr-2* suppressor mutations act by boosting the activity of *paqr-1*. This is not the case: the *nhr-49(et8)* and *cept-1(et10)* mutations still partially suppress the growth defect at 15°C and completely suppress the abnormal tail phenotype of the *paqr-2* mutant at 20°C even when *paqr-1* is mutated (Figure 6A). Similarly, *nhr-49(et8)* and *cept-1(et10)* can completely suppress the grave fertility defect of *paqr-1;paqr-2* at 20°C (Figure 6C). *paqr-1* is therefore not required for the suppression of *paqr-2* phenotypes.

### Lowered PC synthesis suppresses the *paqr-2* cold adaptation defect by promoting fatty acid desaturation

Genetic interaction experiments suggest that *nhr-49(et8)* and *cept-(et10)* likely act on the same target to suppress the *paqr-2* phenotypes. One evidence is the fact that the triple mutant *paqr-2;nhr-49(et8);cept-1(et10)* is viable and grows as well at 15°C as either the *paqr-2;nhr-49(et8)* or *paqr-2;cept-1(et10)* double mutants (Figure 6B). This is consistent with at least two interpretations: 1) the *gof* mutation *nhr-49(et8)* and the *lof* mutation *cept-1(et10)* act on a common target; or 2) *nhr-49(et8)* acts downstream of *cept-1(et10)*. However, we can reject the second of these hypotheses using the *nhr-49(gk405)* null allele since the double mutant *paqr-2;nhr-49(gk405)* is lethal while the triple mutant *paqr-2;nhr-49(gk405); cept-1(et10)* is quite viable (Figure 6A and C). Since *nhr-49* is a well-established promoter of fatty acid desaturation, we tentatively conclude that this is also the essential outcome of the *cept-1(et10)* mutation. We previously showed that a *lof* mutation in *nhr-80*, another nuclear hormone receptor that can activate Δ9-desaturases, partially suppresses the *paqr-2* cold adaptation defect, although this suppression is not nearly as effective as that caused by the *nhr-49 gof* alleles described here [12]. The observations that a *lof* allele of *nhr-80* and a *gof* allele of *nhr-49* can both suppress *paqr-2* mutant phenotypes while the *lof nhr-49(gk405)* is synthetic lethal with the *paqr-2* mutation are at first glance surprising given the documented overlap in function between *nhr-80* and *nhr-49*, for example as activators of Δ9-desaturases. There must therefore be important functional differences between the two genes. NHR-49 has many interaction partners, including NHR-80, and it is possible that the *nhr-80 lof* mutation suppresses the *paqr-2* phenotypes either by allowing more interaction of NHR-49 with its other partners, or by causing the enhanced activation of compensatory pathways, such as SBP-1, to restore desaturase activation. Indeed, *nhr-49* and *nhr-80* mutants are known to have distinct metabolic profiles, with *nhr-49* being involved in a wider range of lipid homeostasis pathways [18], [27].

Walker and co-workers have shown that lower PC levels lead to activation of *sbp*-1/SREBP, and that this regulation is conserved among metazoans [19]. Here, *sbp-1* appears to be particularly important for the survival of *paqr-2* mutants: the *sbp-1(ep79)* partial loss of function allele is lethal in combination with *paqr-2* [12] (Figure 6A). Furthermore, and in contrast to the *paqr-2;nhr-49(gk405)* synthetic lethality, the *paqr-2;sbp-1(ep79)* lethality cannot be suppressed by *cept-1(et10)* nor, incidentally, by *nhr-49(et8)* (Figure 6A). These results are consistent with *cept-1* acting through *sbp-1*, and with *sbp-1* being more important than *nhr-49* for the survival of *paqr-2* mutants.

If *lof* mutations in enzymes of the PC synthesis pathway suppress the cold adaptation defect of the *paqr-2* mutant by increasing Δ9-desaturase activity via *sbp-1*, then inhibiting the desaturases should abolish the *paqr-2* suppression. This is indeed the case: RNAi against *fat-6* or *fat-7* completely desuppresses the 15°C growth of *paqr-2*;*cept-1(et10)* double mutants (Figure 6D). Furthermore, inhibiting *fat-6* or *fat-7* also abolishes the *paqr-2* suppression effects of *nhr-49(et8)* and *hacd-1(et12)* (Figure 6D), suggesting that the effects of all three suppressor mutations converge on FA desaturation. Note that in practice we cannot rely on our RNAi results to distinguish the relative importance of *fat-6* and *fat-7* since these show extensive sequence homology such that the RNAi against either gene probably results in inhibition of both genes.

### The desaturase *fat-7* is regulated by *paqr-2* and its suppressors

**Figure 7:**
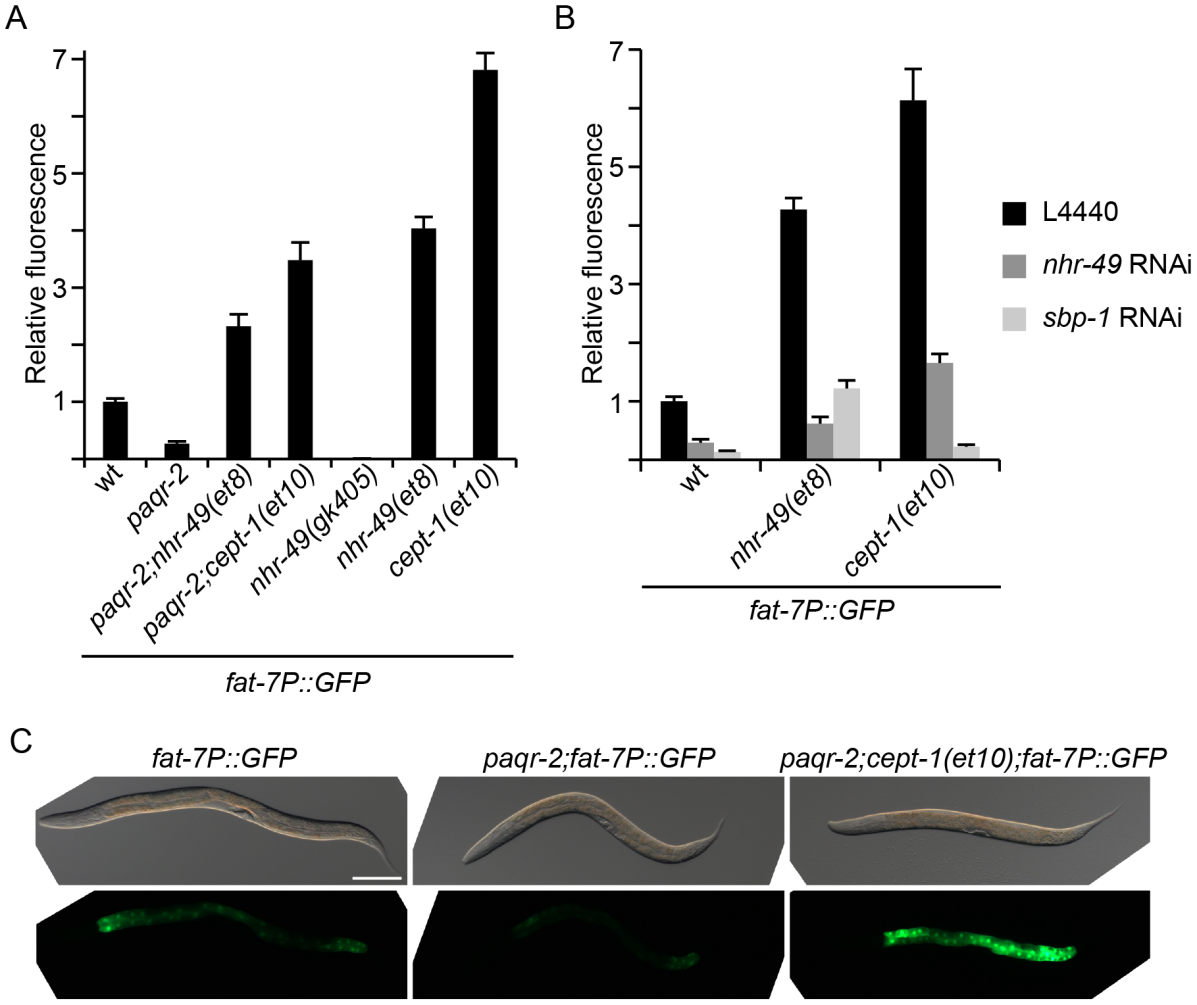
The *fat-7* desaturase is regulated by *paqr-2* and its suppressors. (**A**) The *paqr-2* mutant has decreased expression of *fat-7*, while strains carrying the *paqr-2* suppressors *nhr-49(et8)* or *cept-1(et10)* show a marked increase in *fat-7* expression when compared to control worms (wt). (**B**) The *nhr-49(et8)* and *cept-1(et10)* alleles are dependent on both *nhr-49* and *sbp-1* for the upregulation of *fat-7*. (**C**) Representative images of worms from panel (**A**). Note the strong GFP signal in the *paqr-2;cept-1(et10)* double mutant, especially compared to the low levels of the *paqr-2* single mutant. Scale bar is 100 µm. All strains in this figure carry the *fat-7* promoter GFP reporter in integrated form.

Our observations so far suggest that *paqr-2* normally participates in cold adaptation by regulating the activity of Δ9-desaturases via *sbp-1* (via changes in PC levels) or *nhr-49*. We investigated the possible regulation of Δ9-desaturases by *paqr-2* using a previously published *fat-7* promoter GFP reporter [19]. As anticipated, the *paqr-2* mutant exhibits a marked decrease in *fat-7* expression (Figure 7A and C). Furthermore, the *nhr-49(et8)* and *cept-1(et10)* alleles show a dramatic increase in *fat-7* expression either as single mutants or, importantly, when combined with the *paqr-2* mutation. The *nhr-49(gk405)* has very low *fat-7* expression levels, consistent with its lack of activity. These results clearly show that *paqr-2* is a regulator of *fat-7*, and suggest that mutations that can compensate for the lack of *paqr-2* do so, at least in part, by boosting the activity of *fat-7*. Additionally, by using RNAi to separately knock down *nhr-49* or *sbp-1*, we made the interesting observation that the *nhr-49(et8)* allele depends at least partially on *sbp-1* to cause an increase in *fat-7* expression, and that, conversely, *cept-1(et10)* depends on both *nhr-49* and *sbp-1* to cause an increase in *fat-7* expression. This suggests that there is considerable overlap or even mutual dependency between the *nhr-49* and *cept-1* pathways that regulate *fat-7*.

### Detergents can suppress the *paqr-2* mutant phenotypes

**Figure 8:**
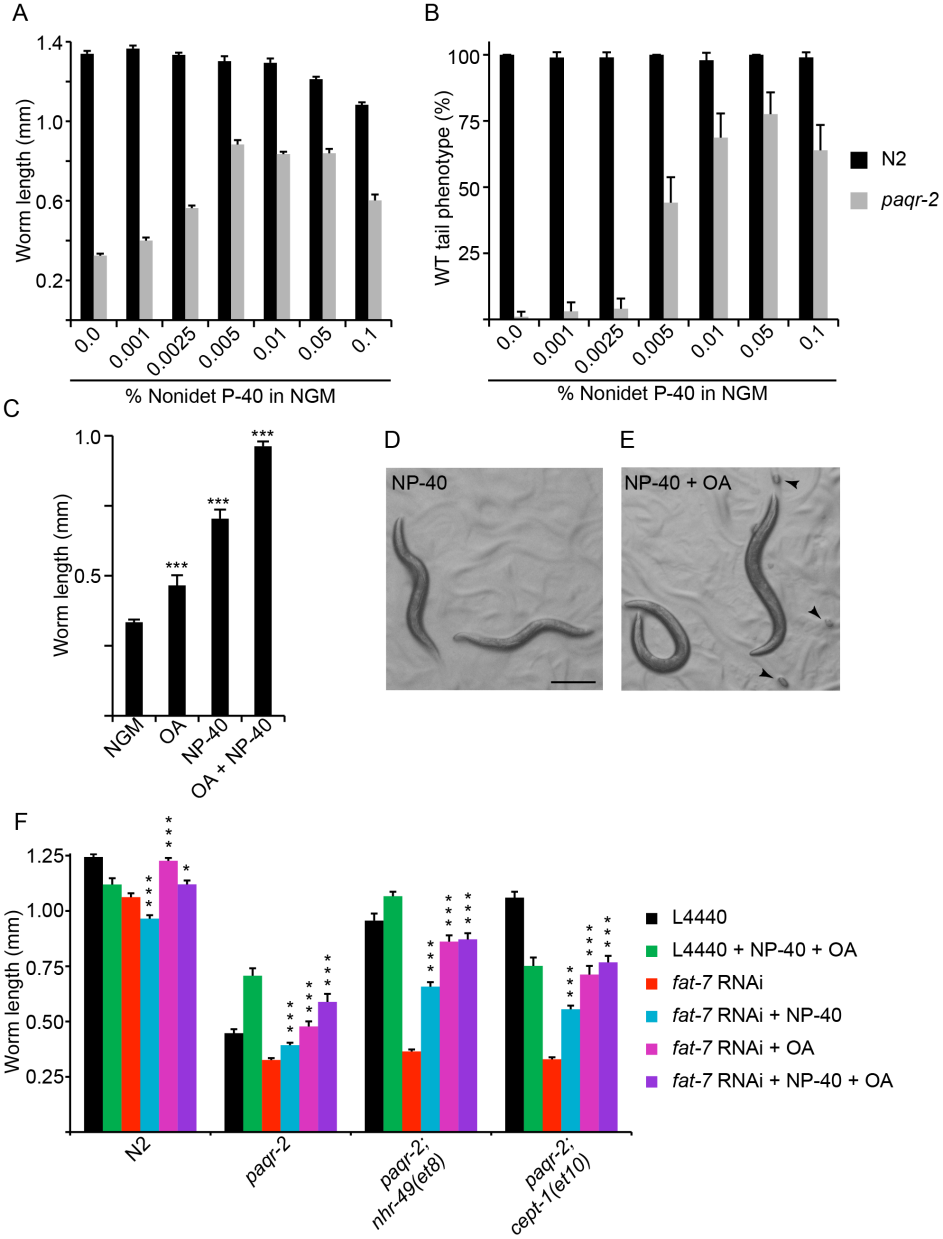
Detergents and oleic acid can suppress the *paqr-2* mutant phenotypes. Low concentrations of Nonidet P-40 (NP-40; **A–B**) suppress the *paqr-2* growth defect at 15°C and the tail tip defect at 20°C. (**C**) 1 mM oleic acid (OA) slightly improves growth of *paqr-2* mutants at 15°C, but not as well as 0.1% Nonidet P-40 (NP-40) or the combination of both. *paqr-2* mutants treated with 0.1% NP-40 alone grow into sterile adults (**D**), and adding also 1 mM oleic acid (OA) restores fertility (**E**; arrowheads indicate eggs). (**F**) RNAi against *fat-7* inhibits the *paqr-2* suppression by *nhr-49(et8)* and *cept-(et10)* but even these double mutants can grow at 15°C when NP-40 or OA are provided exogenously either alone or in combination. Scale bar in D represents 250 µm. *:*p<0.05;* ***: *p*<0.001.

The results described so far suggest that the *paqr-2* mutant cannot adapt to 15°C because of a failure to activate Δ9 desaturases, hence to increase membrane fluidity. Furthermore, since the tail tip morphology defect is correlated with the 15°C growth arrest in most experiments, it is likely that this phenotype also is due to a defect in desaturase regulation. We were intrigued by the possibility of rescuing the *paqr-2* mutant by using detergents at concentrations expected to increase membrane fluidity [28], [29]. Strikingly, we found that small amounts of Nonidet P-40 or Triton X-100 are excellent at rescuing the tail tip morphology at 20°C and the growth at 15°C (Figure 8A–B and Figure S5A–B). The detergents alone did not however provide a full *paqr-2* rescue at 15°C: treated worms grew well, but produced no progeny. It seems likely that the detergents cannot fulfill all biological functions that desaturated FAs have beyond their role in improving membrane fluidity, including possible signaling functions. We therefore tested oleic acid, a monounsaturated FA, as a more biologically natural way of compensating for the reduced levels of unsaturated FAs in the *paqr-2* mutant. On its own, oleic acid produced only a marginal growth rescue at 15°C (Figure 8C and Figure S5C–D). However, the combination of oleic acid and Nonidet P-40 caused complete rescue, allowing the treated *paqr-2* worms to grow and reproduce (Figure 8C–E). Indeed, the combination of 1 mM oleic acid and 0.05% Nonidet P-40 even suppressed the synthetic lethality of *paqr-2 sbp-1(ep79)* worms, allowing double homozygous worm progeny of a *paqr-2 sbp-1(ep79)/+* mother to grow into adults and establish a stable line. Finally, we show that Nonidet P-40 alone, or in combination with oleic acid, suppresses the *paqr-2* growth defect at 15°C even when *fat-7* is inhibited by RNAi (Figure 8F). In other words, exogenously providing either a mild detergent or a desaturated fatty acid can relieve the *paqr-2* mutant from its dependency on increased Δ9-desaturases for growth at a low temperature. These are striking results given our earlier observation that the *paqr-2* suppressor mutations are dependent on the Δ9-desaturases in order to allow growth at 15°C (Figure 6D), and again support the hypothesis that failing to regulate membrane fluidity is a key defect of the *paqr-2* mutant at 15°C or of the *paqr-2 sbp-1* double mutant at 20°C.

## Discussion

### The role of *paqr-2* during cold adaptation

We have shown that mutations in either of two metabolic pathways can suppress the *paqr-2* phenotypes: 1) mutations that reduce phosphatidylcholine synthesis; and 2) mutations that affect FA metabolism. Mutations in either pathway suppress the *paqr-2* phenotypes by augmenting FA desaturation either directly (*gof* alleles of *nhr-49* or *mdt-15*, or *sbp-1* overexpression), or indirectly by reducing PC levels hence upregulating *sbp-1* (*lof* alleles of *sams-1*, *pcyt-1* or *cept-1*), or by increasing the exposure of FA to Δ9 desaturases via increased release from TAGs (*lof* allele of *aak-2*) or inhibition of β-oxidation (*lof* alleles of *ech-7* or *hacd-1*). Increasing the relative abundance of unsaturated FAs in biological membranes increases their fluidity [1]–[3], and this is likely the essential effect of *paqr-2* during cold adaptation.

Several evidences suggest that the pathway outlined in Figure 4 is indeed regulated by *paqr-*2, rather than being a parallel pathway important for cold adaptation. Firstly, all nine *paqr-*2 suppressors isolated in our non-biased screen were part of that pathway. Secondly, all nine suppressors also suppressed a very specific *paqr-2* morphological phenotype that was not screened for, namely the withered tail tip. Thirdly, the convergence of the identified pathway onto Δ9-desaturases is consistent with their established importance during cold adaptation [5], [7]. Fourthly, both the cold adaptation and tail phenotypes of *paqr-2* were corrected by the use of low concentrations of detergents expected to mimic the effects of increasing fatty acid desaturation on membrane fluidity. The present study therefore suggests that *paqr-2* is a transmembrane protein essential for the regulation of Δ9 desaturases during cold adaptation via the modulation of PC levels, hence *sbp-1*. However, the direct point of interaction between PAQR-2 and its target pathway is not immediately apparent, but one interesting hypothesis is that PAQR-2 acts on FA metabolism by regulating PC abundance via an associated phospholipase activity. Indeed, PAQR-2 belongs to a large and diverse protein family of membrane hydrolases that includes *Per1*, a yeast GPI-phospholipase A2 [9].

Others have previously correlated an increase in unsaturated FAs with adaptation to 15°C growth in *C. elegans* [11], and with cold adaptation in many other organisms [1]–[3]. A role for Δ9 desaturases for growth at low temperatures in *C. elegans* has also been documented previously, and it is particularly striking that *fat-6;fat-7* double mutants exhibit a temperature sensitivity similar to the *paqr-2* mutant, growing well at 20°C and poorly at 15°C [5]. Here we add a novel level of regulation by linking *paqr-2*, PCs, *sbp-1* and Δ9 desaturases into a regulatory axis controlling the increase in unsaturated FAs during cold adaptation. At present we do not know whether regulating the activity of Δ9 desaturases is the only essential function of *paqr-2* during cold adaptation.

Our finding that *paqr-2* dependent increases in FA unsaturation is an essential component of the adaptation to 15°C in *C. elegans* may seem to conflict with Murray *et al.* who previously estimated that FA unsaturation changes contribute only about 15% of the cold adaptation response [16]. However, it is important to keep in mind that Murray *et al.* were concerned with survival of *C. elegans* at the acutely lethal temperature of 0°C while we are concerned here with adaptive changes at a low but physiologically acceptable temperature.

### Evolutionary considerations

Could the *paqr-2* homologs play a role during cold adaptation even in homeotherms? Increased serum adiponectin levels have been observed in rats kept at 4°C for 24 hours, with adiponectin mRNA levels becoming elevated in brown adipose tissue (BAT) [30]. Also, human subjects wearing a 10°C liquid-conditioned suit for two hours have shown a near-doubling of circulating adiponectin levels [31]. Furthermore, the AdipoRs are expressed in temperature-sensitive neurons in the brain [32], and adiponectin itself has sequence homology with hibernation-associated plasma proteins in Asian chipmunks and woodchucks [30], [33], which again suggests that it may be part of a protein family that functions during cold adaptation in mammals.

## Materials and Methods

### *C. elegans* cultivation, strains and transgenes

Maintenance of worms were performed as described elsewhere [34]. The wild type reference strain was the *C. elegans* Bristol variety strain, N2. Unless otherwise stated, strains were obtained from the *C. elegans* Genetics Center (CGC; MN, USA) and cultivated at 20°C.

### Western blot

Crowded plates of various *C. elegans* strains were harvested and washed twice in water before lysis by boiling in SDS sample loading buffer. Twenty micrograms of total protein was loaded in each lane, electrophoresed on a SDS polyacrylamide gel, and transferred to nitrocellulose. For Western blot detection of the PAQR-2 protein, a rabbit antiserum was generated by immunizing a rabbit against the peptide PLNVRDWTPADVGL corresponding to the C-terminal amino acids. Crude serum was used at a dilution of 1∶1000. Blotto (5% skim milk powder in TBST) was used for blocking. Antibody dilutions and washes were carried out in TBST. A goat anti-rabbit HRP-conjugated secondary antibody (GE) was used at a final concentration of 1∶2 500 to detect bound primary antibody. Detection of bound antibody was performed using an ECL Detection Kit, as per the manufacturer's instruction (Thermo Scientific).

### Mutagenesis and screen for *paqr-2* suppressors

*paqr-2(tm3410)* worms were mutagenized for 4 hours by incubation in the presence of 0.05 M ethyl methane sulfonate (EMS) according to the standard protocol [34]. The worms were then washed and placed on a culture dish. Two hours later, vigorous hermaphrodite L4 animals were transferred to new culture plates. Five days later, F1 progeny were bleached, washed and their eggs allowed to hatch overnight in M9 (22 mM KH2PO4, 42 mM Na2HPO4, 85.5 mM NaCl and 1 mM MgSO4). The resulting L1 larvae were transferred to new plates, cultivated at 15°C, then screened from day 4 to day 6 to identify *paqr-*2 suppressors able to reproduce at that temperature, which were picked to new plates for further analysis. Presence of the *paqr-2(tm3410)* mutant allele in each suppressor was confirmed using a PCR assay [12].

The isolated suppressor alleles, named *et6* to *et14* were outcrossed 4-to-6 times prior to whole genome sequencing (see below), and 10 times prior to their phenotypic characterization or use in the experiments presented here. Outcrossing was done by mating wild-type N2 males to a suppressor, then crossing the male progeny to *paqr-2* single mutant worms (themselves previously outcrossed 10 times to wild-type worms); progeny from this cross were picked to individual plates and kept at 20°C then screened for homozygozity for *paqr-2* using PCR, followed by testing their F2 progeny for ability to grow at 15°C. Five such cycles were carried out amounting to ten outcrosses (five to N2 worms and five to *paqr-2(tm3410)* worms).

### Whole genome sequencing

The genomes of suppressor mutants that had been outcrossed 4 or 6 times were sequenced to a depth of 25–40x as previously described [22]. The sequencing results were analyzed using the MAPQGene software to produce tables listing all differences between the reference N2 genome and that of the mutants, and sort these differences by criteria such as non-coding substitutions, termination mutations, splice-site mutations, etc. [35]. For each suppressor mutant, one or two hot spots, i.e. small genomic area containing several mutations, were identified, which is in accordance to previous reports [21]. Mutations in the hot spot that were still retained after 10 outcrosses were considered candidate *paqr-2* suppressors and tested experimentally as described in the text.

### Plasmids

The *pNHR-49* plasmids (WT, *et8* and *et13*) were all constructed by amplification of the *nhr-49* promoter, gene and UTR using primers 5′-atccttactggacgccgtctaca-3′ and 5′-gtagtacagtaaccaactttcccgaagt-3′ with the corresponding genotype as template. The resulting ∼6.6 kb PCR products were cloned into *pCR-XL-TOPO* (Invitrogen).

The *pCEPT-1* plasmid was constructed by amplification of the *cept-1* promoter, gene and UTR using primers 5′-gagtttcgataaagtatagcttgtcacga-3′ and 5′- ggttaagcttgttttgtgtaatgcgtagt-3′ and a mixture of N2 worms as template. The resulting ∼6.6 kb PCR product was cloned into *pCR-XL-TOPO*.

The *pHACD-1* plasmid was constructed by amplification of the *hacd-1* promoter, gene and UTR using primers 5′-gaatagttaacaagcgctcagtcatga-3′ and 5′-acagtgtcgccacttcaggactact-3′ and a mixture of N2 worms as template. The resulting ∼4.6 kb PCR product was cloned into *pCR-XL-TOPO*.

The *pMDT-15(et14)* plasmid was constructed by amplification of the *mdt-15* promoter, gene and UTR using primers 5′-gaaattgctcatctatcgggtct-3′ and 5′-atcgcaggaatcggattcgta-3′ and a mixture of *paqr-2;mdt-15(et14)* worms as template. The resulting ∼7.7 kb PCR product was cloned into *pCR-XL-TOPO*.

### Generation of transgenic animals

Plasmids were prepared with a Qiagen miniprep kit (Qiagen) and used with the following concentrations: *pRF4 (rol-6)* of 50 ng/µl, test plasmids of 5–10 ng/µl, and *pBSKS* (Stratagene) of 40–45 ng/µl to a total of 100 ng/µl.

### 15°C growth assays

For confirmation of suppressor alleles or assay of double/triple mutants using the *paqr-2* cold adaptation phenotype at 15°C, synchronized L1s were placed on plates and incubated at 15°C for 5 or 6 days. Pictures were taken using a Zeiss Axiophot microscope and worm lengths measured in ImageJ [36].

### Brood size assay

The brood size experiment confirming *cept-1(et10)* was performed essentially as described [12]. All strains were incubated at 20°C from L1 to 1 day adults before transfer to 15°C.

### *paqr-2* tail tip phenotype assay

For confirmation of suppressor alleles using the withered tail tip phenotype, L4s were picked and scored 24 h later as young adults. n>100 for all tail experiments.

### RNAi feeding

All strains were grown on control L4440 RNAi bacteria for one generation at 20°C, then synchronized and L1s placed onto assay RNAi, incubated at 15°C and scored on day 6. Feeding RNAi clones were from the Ahringer RNAi library.

### RNAi injections

Template for *in vitro* transcription was made by a PCR reaction using a T7 primer (5′-cgtaatacgactcactatag-3′) and feeding RNAi clones as template. *In vitro* transcription was performed using the Riboprobe System-T7 (Promega) and the dsRNAi was purified and injected into *paqr-2* worms. Injected worms were allowed to lay eggs at 20°C and the eggs were transferred to 15°C for scoring of worm length 6 days later. The *ech-7* and *pcyt-1* RNAi clones were from the ORF-RNAi library.

### Quantification of *fat-7::GFP* expression

The *pfat-7::GFP (rtIs30)* carrying strain HA1842 has been described elsewhere [19] and was a kind gift from Amy K. Walker. Quantitative measurements of the GFP intensity was performed on synchronized L4s using the image processing program ImageJ [36]. Presence of the *rtIs30* array in the *nhr-49(gk405)* mutant was assayed by PCR reactions against GFP.

### Lipidomics

Samples were composed of synchronized L4 larvae (one 15 cm diameter plate/sample). Worms were washed 3 times with M9, pelleted and stored at −80°C until analysis. For lipid extraction, the pellet was sonicated for 10 minutes in methanol and then extracted according to published methods [37]. Internal standards were added in the chloroform phase during the extraction. Lipid extracts were evaporated and reconstituted in chloroform∶methanol [1∶2] with 5 mM ammonium acetate. This solution was infused directly (shotgun approach) into a QTRAP 5500 mass spectrometer (ABSciex, Toronto, Canada) equipped with a Nanomate Triversa (Advion Bioscience, Ithaca, NY) as described previously [38]. Phospholipids were measured using multiple precursor ion scanning [39], [40] and triacylglycerols were measured using neutral loss scanning [41]. The data was evaluated using the LipidProfiler software [39].

### Detergents and oleic acid

Detergent or oleic acid plates were made by adding the appropriate amount of 10% Nonidet P-40 substitute, 10% Triton X-100 or 0.5 M oleic acid stock solutions to NGM. Synchronized L1s were placed on detergent plates, incubated at 15°C for 6 days and scored for worm length.

### Statistics

Error bars for worm length measurements show the standard error of the mean, and *t-tests* were used to identify significant differences between worm lengths. Error bars for the frequency of the tail tip defect show the 95% confidence interval determined using *Z-tests*.

## Supporting Information

Figure S1 Structure of the *paqr-2* gene and characterization of the *tm3410* allele. (**A**) Structure of the *paqr-2* transcript (top) and PAQR-2 protein, with the deleted regions in the *tm3410* allele indicated by the red underlines. (**B**) Western blot showing the PAQR-2 band at ∼66 kDa, which is absent in the *paqr-2(tm3410)* mutant but recovered when a *paqr-2* transgene is reintroduced. Bracketed bands in (**B**) indicate degradation PAQR-2 products in the transgenic animals, and other bands are due to non-specific binding of the antibody.
(TIF)

Figure S2 Fertility, brood size and growth rate of the *paqr-2* suppressors. (**A**) Percentage of L1s that grow into fertile adults at three different temperatures. Note that all suppressor mutations permit reproductive growth of the *paqr-2* mutant at 15°C. (**B**) Total self-brood size at 20°C. Note that all suppressor mutations dramatically improve self-brood size, except for *ech-7(et6)*. (**C**) Length of worms grown from the L1 stage at 20°C for different amounts of time. Note that all suppressor mutations improve the growth of the *paqr-2* mutant, except again for *ech-7(et6)*. The figure legend in C also applies to panel A. *: *p*<0.05; **: *p*<0.01; ***: *p*<0.001.
(TIF)

Figure S3 Experimental tests confirming the identity of *paqr-*2 suppressor mutations. (**A**) Injected RNAi against *pcyt-1* or *ech-7* can suppress the *paqr-2* phenotype, suggesting that the *paqr-2* suppressors *et6* and *et9* are *lof* alleles of these genes. (**B**) The *hacd-1(ok2776)* deletion allele is as effective at suppressing the *paqr-2* 15°C growth defect as the *hacd-1(et12)* allele, confirming that *et12* is also a *lof* allele. (**C**) Providing wild-type *cept-1* as a transgene desuppresses the self-brood size defect in *paqr-2;cept-1(et10)*, indicating that *cept-1(et10)* is a *lof* allele. (**D**) Homozygosity for the *cept-1(et10)*, *cept-1(et11)* or *hacd-1(et12)* mutation suppresses the tail defect in *paqr-2* worms, but this phenotype is desuppressed by introducing wild-type *cept-1* or *hacd-1* transgenes in the *paqr-2;cept-1(et10)* and *paqr-2;cept-1(et11)*, or *paqr-2;hacd-1(et12)* strains, respectively. This indicates that *et10*, *et11* and *et12* are *lof* alelles. (**E**) and (**F**) The *sams-1(ok2946)* deletion allele and the *sbp-1* overexpression transgene *epEx141* can also suppress the *paqr-2* 15°C growth defect, respectively. ***: *p*<0.001.
(TIF)

Figure S4 Analysis of PE/PC ratios. (**A**) The PE/PC ratio is elevated in the *paqr-2* mutant and is partially normalized by the *nhr-49(et8)* or *cept-1(et10)* mutations. (**B**) In a separate experiment, again the PE/PC ratio is elevated in the *paqr-2* mutant, unaffected in the *nhr-49(et8)* or *nhr-49(gk405)* mutants, and elevated in the *cept-1(et10)* mutant. The average amounts of lipids recovered per mg of protein from 5 samples for each genotype are also indicated. There is a fair amount of variation in the lipid extraction, as evidenced by the larger error bars for the amount of lipids. However, this did not affect the relative recovery of PEs and PCs, as evidenced by the small error bars for the PE/PC ratios.
(TIF)

Figure S5 The non-ionic detergent Triton X-100 and oleic acid can independently suppress *paqr-2* phenotypes. (**A**) Inclusion of 0.05–0.2% Triton X-100 in the culture plates allows the *paqr-2* mutant to grow at 15°C. (**B**) Including 0.05% Triton X-100 in culture plates allows the *paqr-2* mutant to develop and maintain normal tail tips at 20°C. (**C**) and (**D**) Inclusion of 0.5–2 mM oleic acid in culture plates causes a slight but dose-dependent improvement in the growth of *paqr-2* mutants at 15°C and in the quality of the tail morphology at 20°C, respectively.
(TIF)

Table S1 PC composition (each row indicates the % of total PC that had the indicated number of carbon atoms and double bonds in its two FAs). Red and blue shadings indicate FAs that are significantly elevated or lowered, respectively, compared to the control N2 worms and based on *t*-tests. Note: 17:Δ is cis-9,10-methylenehexadecanoic acid and 19:Δ is cis-11,12-methylene octadecanoic acid.
(XLSX)

Table S2 PE composition (each row indicates the % of total PE that had the indicated number of carbon atoms and double bonds in its two FAs). Red and blue shadings indicate FAs that are significantly elevated or lowered, respectively, compared to the control N2 worms and based on *t*-tests. Note: 17:Δ is cis-9,10-methylenehexadecanoic acid and 19:Δ is cis-11,12-methylene octadecanoic acid.
(XLSX)

Table S3 TAG composition (each row indicates the % of total TAG that had the indicated number of carbon atoms and double bonds in its FAs). Red and blue shadings indicate FAs that are significantly elevated or lowered, respectively, compared to the control N2 worms and based on *t*-tests.
(XLSX)

Table S4 FA composition of PCs, PEs and TAGs. Red and blue shadings indicate FAs that are significantly elevated or lowered, respectively, compared to the control N2 worms and based on *t*-tests. Note: 17:Δ is cis-9,10-methylenehexadecanoic acid and 19:Δ is cis-11,12-methylene octadecanoic acid.
(XLSX)

Table S5 PC composition (each row indicates the % of total PC that had the indicated number of carbon atoms and double bonds in its two FAs). Red and blue shadings indicate FAs that are significantly elevated or lowered, respectively, compared to the control N2 worms and based on *t*-tests. Note: 17:Δ is cis-9,10-methylenehexadecanoic acid and 19:Δ is cis-11,12-methylene octadecanoic acid.
(XLSX)

Table S6 PE composition (each row indicates the % of total PE that had the indicated number of carbon atoms and double bonds in its two FAs). Red and blue shadings indicate FAs that are significantly elevated or lowered, respectively, compared to the control N2 worms and based on *t*-tests. Note: 17:Δ is cis-9,10-methylenehexadecanoic acid and 19:Δ is cis-11,12-methylene octadecanoic acid.
(XLSX)

Table S7 TAG composition (each row indicates the % of total TAG that had the indicated number of carbon atoms and double bonds in its FAs). Red and blue shadings indicate FAs that are significantly elevated or lowered, respectively, compared to the control N2 worms and based on *t*-tests.
(XLSX)

Table S8 FA composition of PCs, PEs and TAGs. Red and blue shadings indicate FAs that are significantly elevated or lowered, respectively, compared to the control N2 worms and based on *t*-tests. Note: 17:Δ is cis-9,10-methylenehexadecanoic acid and 19:Δ is cis-11,12-methylene octadecanoic acid.
(XLSX)
