# Supplementary figures and images for: PAQR-2 Regulates Fatty Acid Desaturation during Cold Adaptation in C. elegans
Source: PLoS Genet. 2013 Sep 12;9(9):e1003801. doi: 10.1371/journal.pgen.1003801 (PMC3772066; doi:10.1371/journal.pgen.1003801)

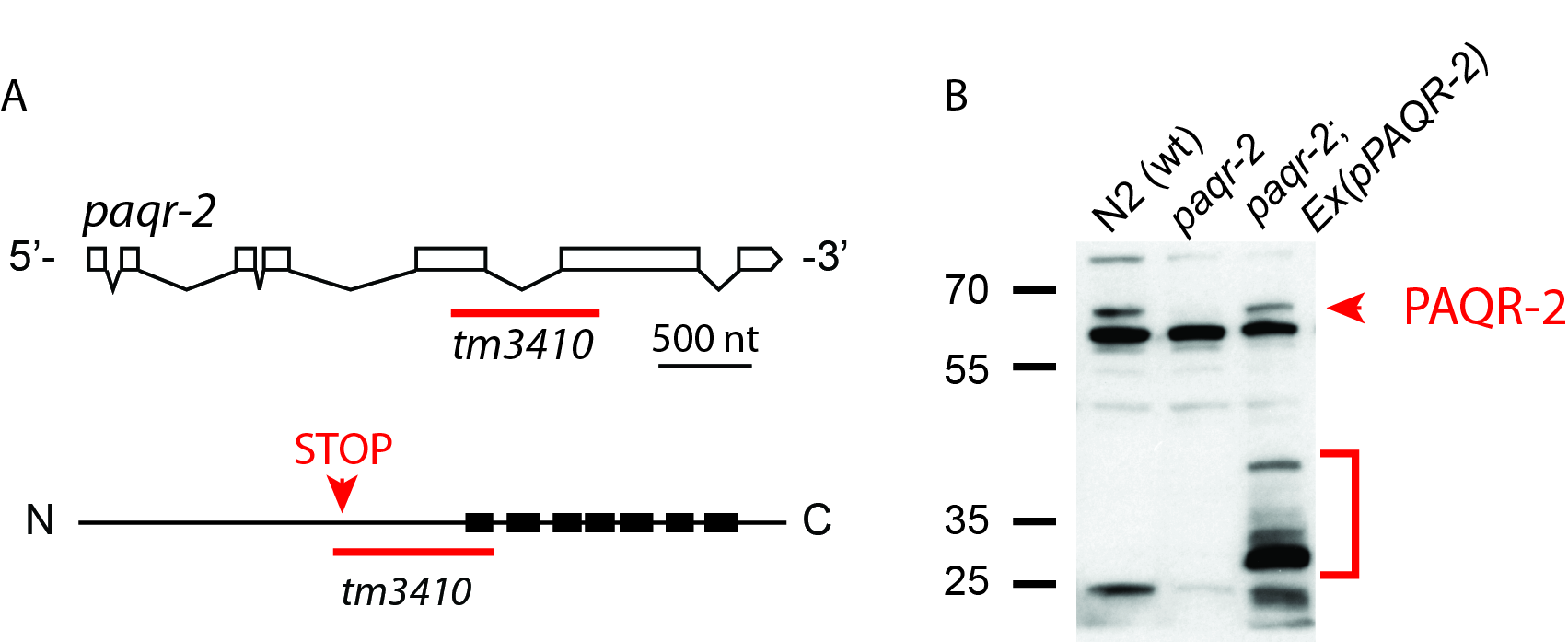

Supplement: Figure S1 — Structure of the paqr-2 gene and characterization of the tm3410 allele. (A) Structure of the paqr-2 transcript (top) and PAQR-2 protein, with the deleted regions in the tm3410 allele indicated by the red underlines. (B) Western blot showing the PAQR-2 band at ∼66 kDa, which is absent in the paqr-2(tm3410) mutant but recovered when a paqr-2 transgene is reintroduced. Bracketed bands in (B) indicate degradation PAQR-2 products in the transgenic animals, and other bands are due to non-specific binding of the antibody. (TIF) [file pgen.1003801.s001.tif]

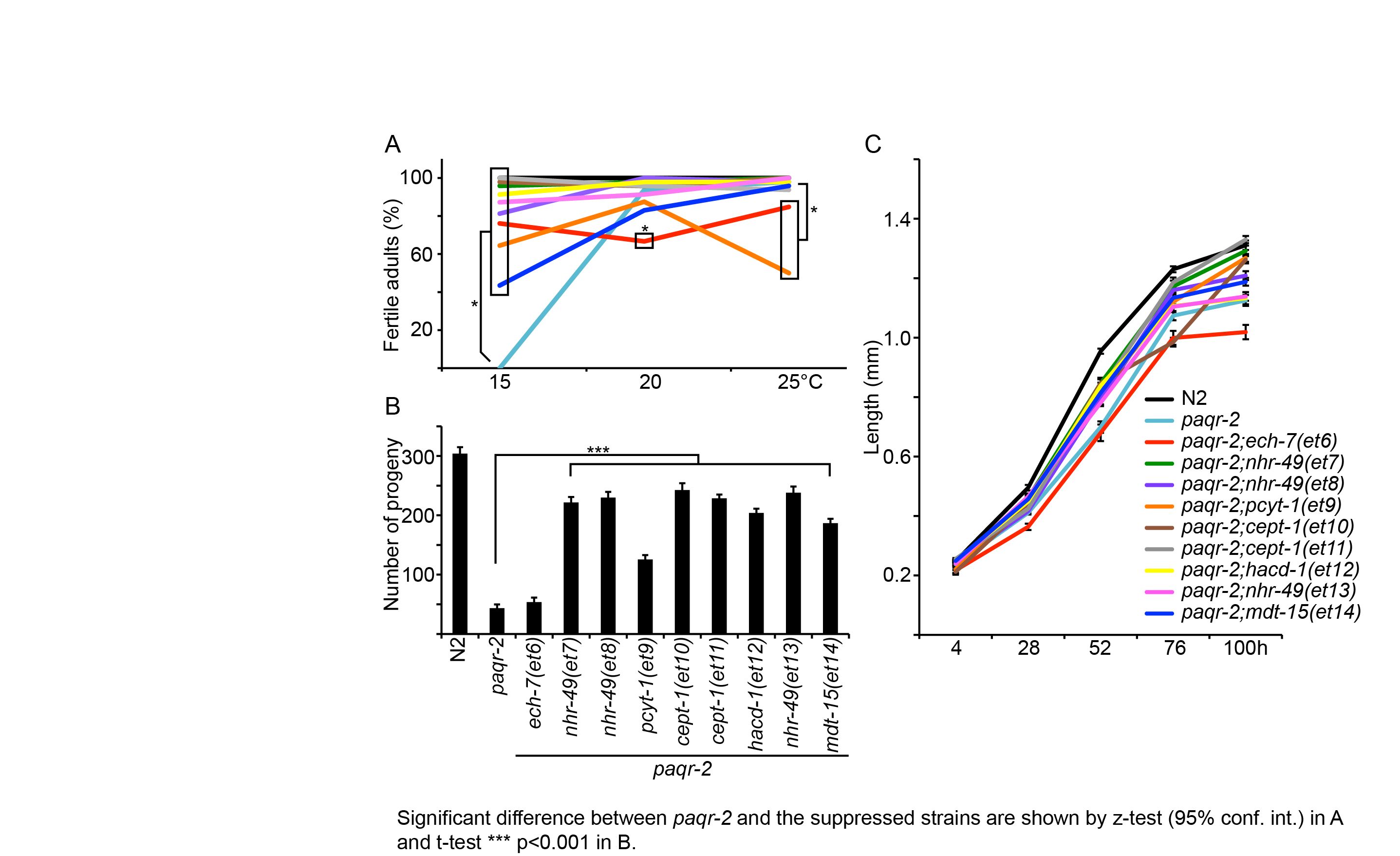

Supplement: Figure S2 — Fertility, brood size and growth rate of the paqr-2 suppressors. (A) Percentage of L1s that grow into fertile adults at three different temperatures. Note that all suppressor mutations permit reproductive growth of the paqr-2 mutant at 15°C. (B) Total self-brood size at 20°C. Note that all suppressor mutations dramatically improve self-brood size, except for ech-7(et6). (C) Length of worms grown from the L1 stage at 20°C for different amounts of time. Note that all suppressor mutations improve the growth of the paqr-2 mutant, except again for ech-7(et6). The figure legend in C also applies to panel A. *: p<0.05; **: p<0.01; ***: p<0.001. (TIF) [file pgen.1003801.s002.tif]

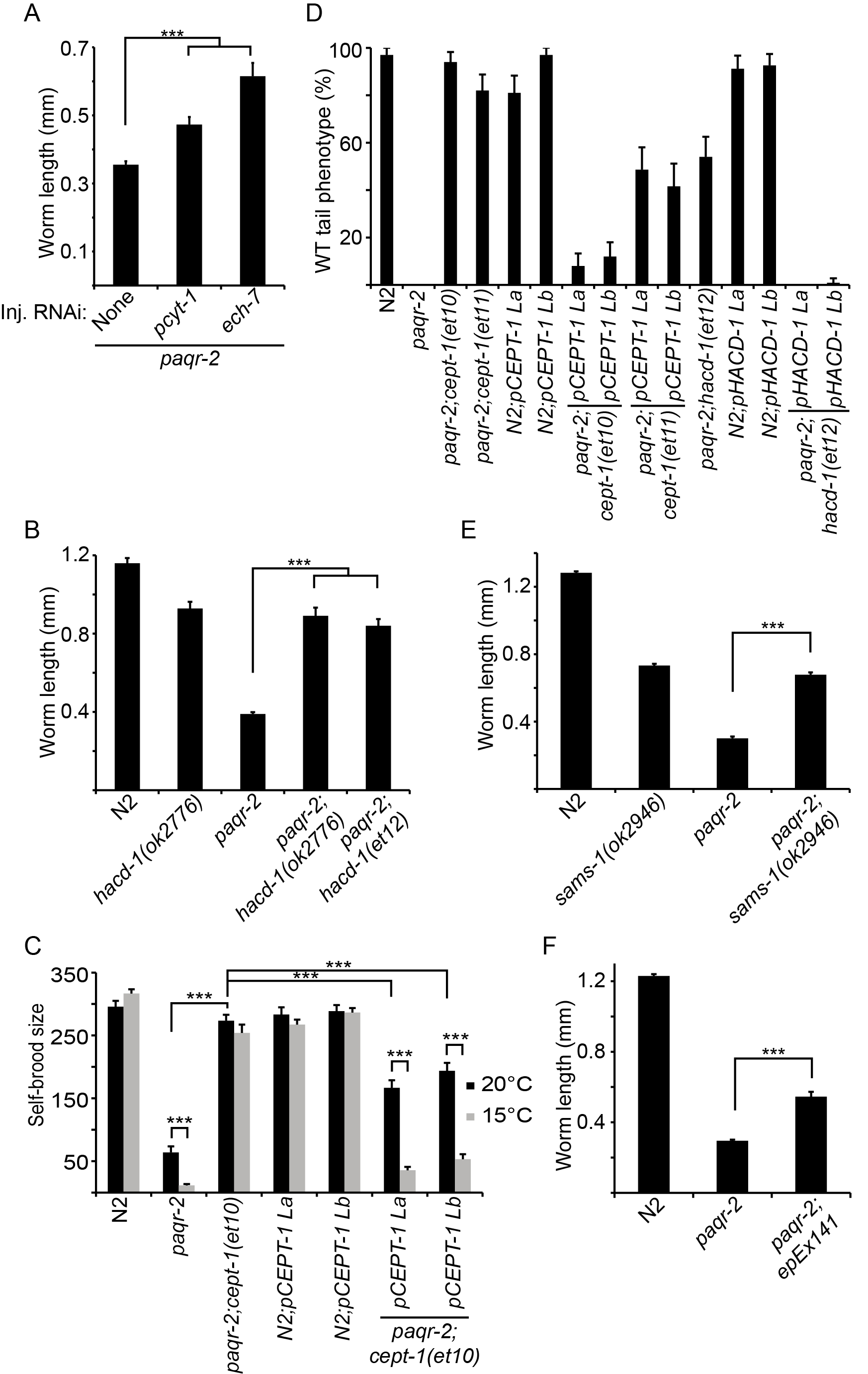

Supplement: Figure S3 — Experimental tests confirming the identity of paqr-2 suppressor mutations. (A) Injected RNAi against pcyt-1 or ech-7 can suppress the paqr-2 phenotype, suggesting that the paqr-2 suppressors et6 and et9 are lof alleles of these genes. (B) The hacd-1(ok2776) deletion allele is as effective at suppressing the paqr-2 15°C growth defect as the hacd-1(et12) allele, confirming that et12 is also a lof allele. (C) Providing wild-type cept-1 as a transgene desuppresses the self-brood size defect in paqr-2;cept-1(et10), indicating that cept-1(et10) is a lof allele. (D) Homozygosity for the cept-1(et10), cept-1(et11) or hacd-1(et12) mutation suppresses the tail defect in paqr-2 worms, but this phenotype is desuppressed by introducing wild-type cept-1 or hacd-1 transgenes in the paqr-2;cept-1(et10) and paqr-2;cept-1(et11), or paqr-2;hacd-1(et12) strains, respectively. This indicates that et10, et11 and et12 are lof alelles. (E) and (F) The sams-1(ok2946) deletion allele and the sbp-1 overexpression transgene epEx141 can also suppress the paqr-2 15°C growth defect, respectively. ***: p<0.001. (TIF) [file pgen.1003801.s003.tif]

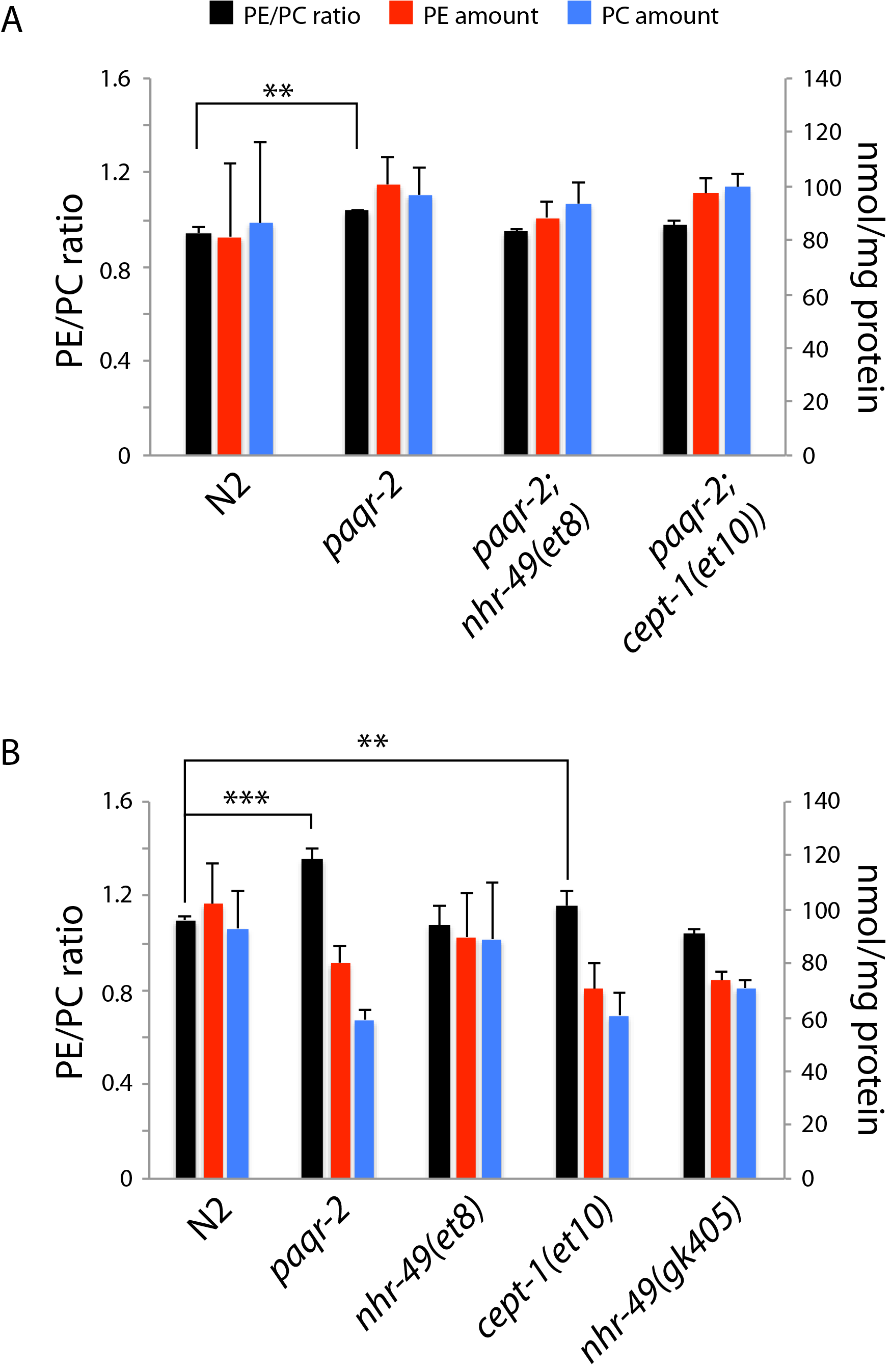

Supplement: Figure S4 — Analysis of PE/PC ratios. (A) The PE/PC ratio is elevated in the paqr-2 mutant and is partially normalized by the nhr-49(et8) or cept-1(et10) mutations. (B) In a separate experiment, again the PE/PC ratio is elevated in the paqr-2 mutant, unaffected in the nhr-49(et8) or nhr-49(gk405) mutants, and elevated in the cept-1(et10) mutant. The average amounts of lipids recovered per mg of protein from 5 samples for each genotype are also indicated. There is a fair amount of variation in the lipid extraction, as evidenced by the larger error bars for the amount of lipids. However, this did not affect the relative recovery of PEs and PCs, as evidenced by the small error bars for the PE/PC ratios. (TIF) [file pgen.1003801.s004.tif]

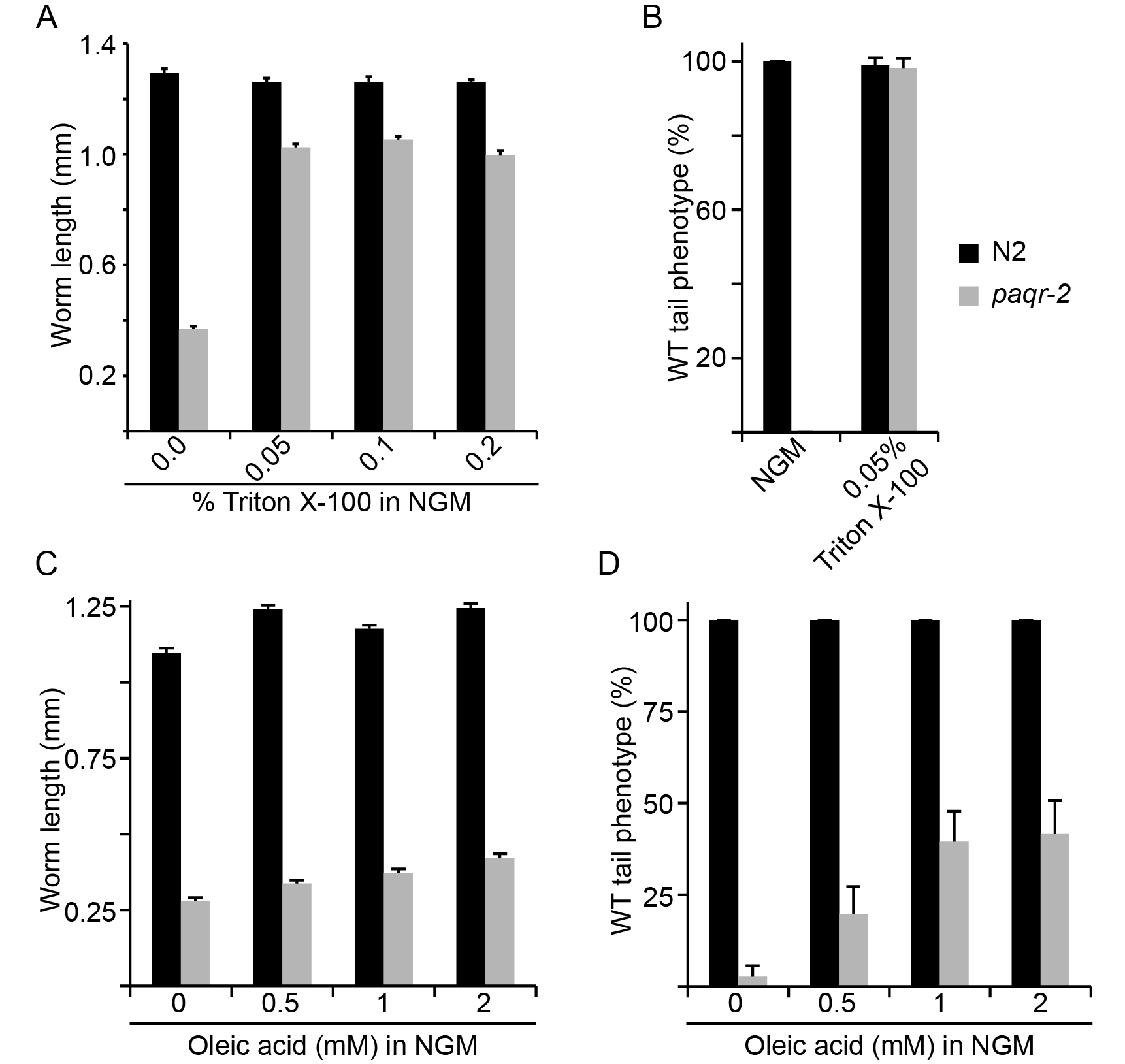

Supplement: Figure S5 — The non-ionic detergent Triton X-100 and oleic acid can independently suppress paqr-2 phenotypes. (A) Inclusion of 0.05–0.2% Triton X-100 in the culture plates allows the paqr-2 mutant to grow at 15°C. (B) Including 0.05% Triton X-100 in culture plates allows the paqr-2 mutant to develop and maintain normal tail tips at 20°C. (C) and (D) Inclusion of 0.5–2 mM oleic acid in culture plates causes a slight but dose-dependent improvement in the growth of paqr-2 mutants at 15°C and in the quality of the tail morphology at 20°C, respectively. (TIF) [file pgen.1003801.s005.tif]
